# The Alleged Increase of Heart Disease

**Published:** 1895-07-06

**Authors:** 


### The Alleged Increase of Heart Disease.

A cursory investigation of the statistics published year by year by the Registrar-General elicits facts which, if not carefully compared with other details to be obtained from the same source, would probably lead to very erroneous conclusions. One of the most striking points, for example, shown by a comparison of the death rates from various causes during a long series of years is the steady increase in the proportionate mortality caused by cancer and by diseases of the circulatory system. Diseases of the nervous system also seem to have increased in frequency. It certainly is a startling thing to find that whereas the mean annual death-rate from diseases of the heart and circulatory system was only 1·00 per thousand living between 1861 and 1865, it had risen to 1·69 in the years between 1886 and 1890; and those who incline to take gloomy views may with a certain show of reason declaim against the hollowness of our boasted progress if the sanitary improvements of the past thirty years have but had the effect of altering the manner in which we die, saving us from fevers only to kill us off by the cruel hand of heart disease.

Before, however, we accept so depressing a conclusion, we must find an answer to another question, viz., who are the victims of heart disease on the one hand and of zymotic diseases on the other. A very little inquiry shows that the main sufferers from these two classes of disorders are not to be found in the same groups of ages, and that whereas the fevers mostly kill the young, heart diseases are modes of death especially common among the elderly; and thus we see that so far from the increase of deaths from heart disease, which is taking place coincidently with a lowered mortality from fevers, being a mere matter of robbing Peter to pay Paul, it is to a considerable extent a substitution of death at the end of life for death at the beginning—not an undesirable change on the hypothesis that life is worth living.

The figures which lead to this conclusion are worth quoting. The following table, condensed from Whitelegge's Hygiene, gives the mean annual death-rates at different ages per thousand living at each age:—

| Ages. | 1851-60. | 1871-80 | Ages. | 1851-60. | 1871-80 |
|---|---|---|---|---|---|
| 0 5 ... | 67·6 ... | 63·1 | 35 45 ... | 12·3 ... | 12·6 |
| 5-10 ... | 8 5 ... | 6·4 | 45-55 ... | 16·5 ... | 17·7 |
| 10-15 ... | 5 0 ... | 6 4 | 55-65 ... | 28·9 ... | 31·5 |
| 15-20 ... | 7·0 ... | 5·3 | 65-75 ... | 61·7 ... | 64·9 |
| 20-25 ... | 8·7 ... | 7·0 | Over 75 ... | 159·8 ... | 161·6 |
| 25-35 ... | 9 8 ... | 8·9 | | | |

This table shows that in the later of these two periods people over 35 died in greater numbers than they used to do twenty years before, but they also show up to that age the mortality was considerably less, so that the excess found in the later years of life is largely due to the dying off of people who in former times would have died at younger ages.

If now we inquire what sort of people are mostly killed by zymotic diseases on the one hand, and by circulatory diseases on the other, we may express the facts by the following table:—

Mean Annual Death-rates from Zymotic Diseases (excluding Diarrhœa) and from Diseases of the Circulatory System per Thousand Living at each Age-period.

| Age period ... | 0-5 | 5-10 | 10-15 | 15-20 | 20-25 | 25-35 |
|---|---|---|---|---|---|---|
| Zymotic diseases | 11·36 | 2·96 | 1·03 | 00·88 | 00·90 | 00·73 |
| Diseases of circulatory system | 00·09 | 00·14 | 00·24 | 00·30 | 00·34 | 00·62 |
| **Age period ...** | **35-45** | **45-55** | **55-65** | **65-75** | **75** | |
| Zymotic diseases | 00·59 | 00·53 | 00·56 | 00·62 | 00·55 | |
| Diseases of circulatory system | 1·31 | 2·27 | 4·81 | 9 48 | 12·09 | |

From this it is plain that, whereas zymotic diseases steadily decrease in their importance as causes of death, as age advances, diseases of the circulation as steadily increase; and when these two tables are studied together they readily explain the third, which gives the number of deaths from diseases of the circulatory system in different groups of years, from 1861 to 1890, as follows:—

| Periods. | 1861-65 | 1866-70 | 1871-75 | 1876-80 | 1881-85 | 1886-90 |
|---|---|---|---|---|---|---|
| Deaths from diseases of the circulatory system per 1,000 living ... | 1·00 | 1·10 | 1·26 | 1·42 | 1·47 | 1·69 |

As it stands, this steady rise in the frequency of deaths from diseases of the circulatory system is sufficiently startling, but there can be no doubt that it is partly explicable by the fact that this is but a mode of death among the elderly and those past middle life. There are, however, other causes at work which also tend to explain this apparent increase of a particular class of maladies, and first among them must be placed greater accuracy of diagnosis.

There are four groups of diseases among which it may often be a matter of doubt where to enter a particular death. They are, if one may so say, interchangeable. Many a case which might truly be returned as bronchitis or as a disease of the digestive system, finds its way, under more careful diagnosis, to its proper place as a disease of heart or kidney; and it is a curious fact that, although all these are diseases of advancing life, they have not all increased in equal proportions, and that those have increased the most into which the others tend to be transferred in consequence of the more accurate diagnosis which is now expected from medical practitioners, heart diseases and urinary diseases having increased far more than affections either of the stomach or the respiratory organs. If the mean death-rates from these four diseases are added together, there still remains an increase in the mortality caused by them as years go on, but it is an increase much more in proportion to the growing mortality which the preceding tables show to be taking place in later life than is the extraordinary advance in the mortality from diseases of the circulatory system which, according to the returns, has occurred during the past thirty years.

